# The Effect of Calcium hydroxide, Glass Ionomer and light cured resin modified calcium silicate on viability, proliferation and differentiation of stem cells from human exfoliated deciduous teeth

**DOI:** 10.1186/s12903-023-03429-6

**Published:** 2023-10-06

**Authors:** Rana Ahmed Shalaby, Amr Mahmoud Abdel-Aziz, Laila Ahmed Rashed, Mohamed Zayed Radwan

**Affiliations:** 1 Department of Pediatric Dentistry and Public Health, Zagazig University, Zagazig, Egypt; 2 Department of Pediatric Dentistry and Public Health, Ain Shams University, Cairo, Egypt; 3 Department of Medical Biochemistry and Molecular Biology, Cairo University, Cairo, Egypt

**Keywords:** SHEDs, Calcium hydroxide, Glass ionomer cement, TheraCal LC, Proliferation, Differentiation, Primary teeth, Vital pulp therapy

## Abstract

**Background:**

Vital pulp therapy, based on the use of stem cells, has promising research and therapeutic applications in dentistry. It is essential to understand the direct effect of capping materials on the dental pulp stem cells of primary teeth, which contribute to the healing powers of the tooth. The aim of this study is to evaluate the effect of different capping materials (Calcium Hydroxide (DyCal®) – Glass Ionomer (Fuji IX®) and light-cured resin modified calcium silicate (TheraCal LC®)) on the viability, proliferation, and differentiation of stem cells from human exfoliated deciduous teeth (SHEDs).

**Methods:**

SHEDs were isolated from extracted primary teeth, then divided into four groups and each of the capping materials were applied to the stem cells as follows: group I the controls, group II with Ca(OH)2, group III with the GIC, and group IV with the Theracal LC. For all groups assessment of viability and proliferation rate was done using the MTT cell proliferation assay. Also, Differentiation was evaluated by measuring the gene expression of Alkaline phosphatase enzyme activity (ALP) and Dentin matrix protein-1 (DMP1) through quantitative real-time PCR. Morphological assessment was conducted using Alizarin Red S staining. All evaluations were performed after 7 and 14 days of culture.

**Results:**

TheraCal LC showed the highest values of proliferation, which was significant only compared to the control group after 2 weeks (*p* = 0.012). After one week, TheraCal LC showed the highest significant values of ALP and DMP1 compared to all other groups (*p* < 0.001).

**Conclusion:**

The three materials under study are biocompatible, maintain viability, and stimulate the proliferation and differentiation of SHEDs. However, TheraCal LC allows better proliferation of SHEDs than Dycal Ca(OH)2 and Fuji IX GIC.

**Supplementary Information:**

The online version contains supplementary material available at 10.1186/s12903-023-03429-6.

## Introduction

Stem cells are undifferentiated, immature cells capable of developing into various cell types through differentiation. Oral tissues have been identified as a rich source of stem cells, offering accessibility for dentists [1].

Dental stem cells can be isolated from various dental structures, including human dental pulp tissue of permanent teeth (hDPSCs), pulp tissue of human exfoliated deciduous teeth (SHEDs), apical papilla (SCAPs), dental follicle tissue (DFSCs), periodontal ligament (PDLSCs), and gingiva tissue (GMSCs). SHEDs, DPSCs, and SCAPs are categorized as dental pulp-related stem cells, while PDLSCs and DFPCs are considered periodontal-related stem cells [2–4].

Stem cells from human exfoliated deciduous teeth (SHEDs) were first identified by Miura et al. in 2003 and possess advantages such as easy accessibility, minimally invasive retrieval, high proliferation potency, and multipotent mesenchymal stem cell properties, besides the ability to differentiate into dentin-pulp complex cells [5]. Hence, SHED cells were selected as the stem cells of choice for investigating different pulp capping materials invitro which are frequently utilized in pediatric dentistry and to assess their impact on the dentin-pulp complex.

Vital pulp therapy is defined as a treatment which aims to preserve and maintain the dental pulp connective tissue that has been compromised, Following the diagnosis of deep carious/traumatic pulp injuries of primary teeth, vital pulp therapy may be clinically performed by applying a capping material directly onto the pulp tissue to allow pulp healing and mineralized tissue barrier formation [6]. Incase of Indirect pulp therapy (IPT), many materials can be used may include glass ionomer cement (GIC), resin-modified glass ionomer cement (RMGIC), adhesive resin, calcium hydroxide, mineral trioxide aggregate (MTA), and silicate cement. Also, several materials have been proposed for direct pulp capping in primary teeth; including mineral trioxide aggregate (MTA), calcium hydroxide, bioactive glass, calcium enriched mixture (CEM) cement and enamel matrix derivative [7].

A Systematic Review and Meta-analysis conducted in 2017 also stated that the highest level of success and quality of evidence supported IPT in primary teeth after 24-months. Direct pulp capping (DPC) showed similar success rates to IPT, but the quality of the evidence was lower [7, 8].

Capping materials are directly applied to the pulp tissue to promote pulp healing and dentine formation. These capping materials should exhibit good biocompatibility, induce minimal pulpal inflammation, and facilitate hard tissue barrier formation as they interact with the pulp's stem cells [9]. Evaluating the biological changes occurring in teeth, such as tooth pulp reactions to restorative procedures and materials, is essential to promote healing and preserve the vitality of the dental pulp [10]. In vitro and in vivo studies on capping materials have aided in material selection for achieving successful treatment outcomes [11–13].

Numerous dental materials are used in restoring of primary teeth, among them is the Calcium Hydroxide (CaOH_2_) which is considered the gold standard when it comes to pulp capping known for its capacity to encourage tertiary dentinogenesis while also exhibiting an antimicrobial feature. CaOH_2_’ s antibacterial capabilities have been meticulously listed by Estrela et al. (1998) [14], including the ability to hydrolyze bacterial cell wall lipopolysaccharides, neutralise bacterial endotoxins, and reduce anaerobic organisms via carbon dioxide absorption [14].

Moreover, Glass Ionomer cement has been widely used in pediatric dentistry and its main advantage beside chemical bonding is the long-term antibacterial effect due to fluoride release [15]. Recently introduced GICs have acceptable wear resistance and a tooth-like coefficient of thermal expansion [16].

The desired properties of a capping material have been exhibited by a subgroup of bioactive materials namely hydraulic calcium silicate– based cements (HCSCs), The desirable properties of silicate-based materials led to the development of new material compositions, such as resin-modified calcium silicate–based materials. Among them, TheraCal LC (ThLC; Bisco Inc, Schamburg, IL) which was introduced as a light curing material for vital pulp therapy, combining the desirable properties of the silicate-based component and the superior handling of resin [17].

These materials may trigger Dentinogensis which begins with the formation of the organic predentin then followed by its subsequent mineralization. This organic matrix has a mineralization regulatory capacity [18]. Collagen represents about 90% of the dentin matrix protein (DMP) where collagen molecules are arranged into fibers. These fibers form collagenous network in which the mineral crystals are deposited [19].

The alkaline phosphatase (ALP) enzyme plays a critical role in facilitating mineral deposition and serves as a significant marker during the early stages of differentiation, with its activity escalating as odontoblasts progress in their differentiation journey. This enzyme's involvement extends beyond mere phosphate ion transportation towards the mineralization front in developing dental tissues; it also participates in the phosphorylation of organic macromolecules [20, 21].

Consequently, both the activity of the alkaline phosphatase enzyme (ALP) and the expression of the Dentin matrix protein (DMP-1) gene serve as dependable methods for evaluating the capability of stem cells to differentiate and construct structures resembling dentin.

This study aims to assess the effects of three capping materials on stem cells from human exfoliated deciduous teeth, specifically investigating their impact on cell proliferation and differentiation, thus providing insights for the proper use of these materials to preserve the pulp of primary teeth. The null hypothesis of the study is that there is no difference between the different capping materials used and the control group in terms of cell proliferation and differentiation.

## Materials and methods

The procedure was simply explained for each patient and informed written consents were taken from the parents. Ethics approval was obtained from the ethical committee of research, Faculty of Dentistry, Ain Shams University on May 2018 (Approval no. FDASU-Rec D051802) This was performed in line with the principles of the Declaration of Helsinki.

## Teeth collection and transportation

A total of Eight Primary teeth from different patients were collected at the outpatient clinic of the Department of Pediatric Dentistry and Dental Public Health, Faculty of Dentistry, Ain Shams University, Cairo, Egypt, from children aged 7–12 years. Teeth indicated for extraction due to normal shedding, early removal for orthodontic purposes, or over-retention were selected. The extracted teeth were required to be sound, with at least one-third of the root remaining and red-colored pulps. Patients with systemic or oral infections were excluded, and only samples from medically healthy individuals were included.

The extracted teeth were immediately kept in a sterile tube containing Dulbecco’s Modified Eagle’s Medium (DMEM) supplemented with 20% fetal bovine serum (FBS). To avoid bacterial infection and contamination of the specimens, penicillin (500 U/mL), streptomycin (500 µg/mL), and amphotericin B (1.25 µg/mL) were added to the transport medium. The teeth were then transported on ice in a closed box to the lab at the unit of Biochemistry and Molecular Biology, Department of Biochemistry, Faculty of Medicine, Cairo University, for stem cell isolation.

## Isolation of mesenchymal stem cells (SHED)

The extracted deciduous teeth were split open, and the pulp was gently removed using a sterile endodontic H-file. The SHED isolation and all the cell culture procedures were performed in a sterile environment in a biological safety cabinet. The pulp was washed three times with phosphate-buffered saline (PBS), digested using collagenase type II (Sigma Aldrich, USA), centrifuged, and then incubated at 37℃ and 5% CO2 in an incubator. On the second day, the cell medium was replaced with 10 mL of fresh complete medium. By day 3, the growth medium was removed, and adherent cells were washed twice with 1 × PBS and detached by incubating the cells with 2 mL of trypsin (2.5 g/L)/EDTA (1 g/L) for 4–5 min at 37℃. The cells were then centrifuged at 1000 rpm for 5–10 min at 17℃ in a pre-cooled centrifuge. After removing the supernatant, the cells were resuspended by adding 4 mL of complete medium, and the cell suspension was passed several times through a pipette to disaggregate cell clumps. Fourth passages of the SHEDs were used, the cells were finally stored at 4℃ until use.

### Cell surface marker expression analysis, characterization using flow cytometry (Fig. 1)

**Fig. 1:**
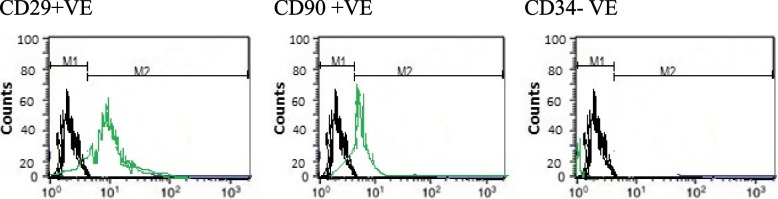
Flow cytometry analysis of stem cell surface markers

After a brief centrifugation, cells were re-suspended in wash buffer (BD Biosciences, Germany). Three hundred ml of cell suspension was incubated with antibodies against CD29, CD34 and CD90 conjugated with Allophycocyanin (APC), Cyanine 5 (CY5), Phycoerythrin (PE) and Fluorescein isothiocyanate (FITC) dyes respectively for 45 min at room temperature. Flow cytometry was performed on a FACS Calibur (BD Biosciences, Germany) and Cell Quest software was used for analysis.

Immunophenotype of stem cells was examined by flow cytometry. BM-MSCs cells were negative for the hematopoietic marker CD34, while strongly positive for stem cell specific markers including CD29 and CD90 (Fig. 1).

## Materials preparation

Split Teflon molds were prepared with a diameter of 3 mm and a thickness of 1 mm. A total of 24 molds were prepared and disinfected before use. All materials were prepared following the manufacturers’ instructions. The DyCal® Calcium hydroxide (DY; Dentsply, Japan), Fuji IX® Glass ionomer (GI; GC, Japan) specimens were mixed using a sterile glass slab and a spatula, while TheraCal LC® was supplied by the manufacturer in pre-mixed syringes and required no preparation before use. All materials were placed in split Teflon molds (8 molds for each material) and manipulated with a sterile probe to ensure a smooth specimen that is free from air bubbles (Table 1). Each group of specimens was given a random number and blindly handed to the lab assessors for application on the isolated SHEDs to prevent bias.

**Table 1:** Group distribution and the materials used

groups	Materials used	Trade name	manufacturer	Number of specimens
Group I (Control group)	-	-	-	*n* = 8
Group II	Ca(OH)2	The DyCal®	DY; Dentsply, Japan	*n* = 8
Group III	GIC	Fuji IX®	GI; GC, Japan	*n* = 8
Group IV	Resin modified calcium silicate	TheraCal LC®	BISCO Inc	*n* = 8

## Viability and proliferation assessment

Cell viability and proliferation were determined using 3-(4,5-dimethylthiazol-2-yl)-2,5-diphenyltetrazolium bromide (MTT) [22] (Trivigen Inc., Gaithersburg, MD, USA) assays, according to the manufacturer's instructions. The cells were cultured in 100 µL of culture medium in a flat-bottomed 96-well plate (tissue culture grade). The MTT reagent (Cat# 4890–25-01) was added (10 µL per well), and the plate was incubated for 2 to 12 h to allow for intracellular reduction of the soluble yellow MTT to the insoluble purple formazan dye. Detergent reagent (Cat# 4890–25-02) solution was added to each well to solubilize the formazan dye prior to measuring the absorbance of each sample in a microplate reader at 570 nm. The absorbance was correlated with the cell number/ml. The cell number that yielded an absorbance of 0.75—1.25 was selected. Eight wells were used for each material.

Cell proliferation was assessed as the percentage of cell proliferation compared to untreated Mesenchymal Stem cells (SHEDs) as control cells. It was evaluated after 7 and 14 days for each material.

## Odontogenic differentiation

Odontogenic differentiation was induced by incubating the confluent monolayers with DMEM supplemented with 10% FBS and 50 μg/ml of L-ascorbic acid 2-phosphate (Wako Pure Chemical Industries, Ltd, Osaka, Japan). The media were changed twice a week.

A. QRT-PCR gene expression of Alkaline phosphatase enzyme and Dentin matrix protein in SHED cells: [23]

RNA extraction from SHED cells was performed, and the yield of total RNA obtained was determined spectrophotometrically at 260 nm. Then, the total RNA (0.5–2 μg) was used for cDNA conversion using a high-capacity cDNA reverse transcription kit (#K1621, Fermentas, USA).Real-time qPCR using SYBR Green I amplification and analysis was performed using an Applied Biosystem with software version 3.1 (StepOne™, USA). The qPCR assay with the primer sets was optimized at the annealing temperature (Table 2).

**Table 2:** Primer Gene Sequence 5′ 3’ of the studied genes ALP and DMP1

	**SEQUENCE**	**Tm**	**Product length**	**GENE BANK ACCESSION NUMBER**
ALP	Forward primer: CACTGCGGACCATTCCCACGTCTT Reverse primer: GCGCCTGGTAGTTGTTGTGAGCATA	60c	301bp	NM 00112750.4
DMP1	Forward primer: GCATCAGGTGGCCAAAGTAT Reverse primer: GAAATCCCATGCAACGTTCT	58c	155 bp	XM 019962170.1
β-actin gene	Forward primer: GCCCTGGACTTTGAGAATGAGAT Reverse primer: TCAGCAATACCAGGGTACA	55c	124 bp	XM 033971831.2

### Primers sequence of studied genes: primer gene sequence 5′ 3’ (Table 2)

Gene expression of ALP enzyme activity and DMP 1 was evaluated twice for each material under the study, after 7 and 14 days.

B. Differentiation by Alizarin Red S staining

In this study, Alizarin Red stain was utilized in a biochemical assay to examine the presence of calcific deposits inside and outside the cells of an odontogenic lineage. The mineralized nodules were stained and observed using an inverted phase contrast light microscope, and digital micrographs were captured at two time points: day 7 and day 14.

### Statistical analysis

The data were presented as mean values along with their standard deviations (SD). Normality of the data was assessed using the Kolmogorov–Smirnov and Shapiro–Wilk tests, indicating that the data followed a normal distribution. Consequently, a One-Way ANOVA was employed to compare the tested groups, followed by the Tukey HSD test for pairwise comparisons. To compare the different follow-up periods, a dependent t-test was utilized. The significance level was set at P ≤ 0.05. The statistical analysis was performed using IBM SPSS Statistics software, Version 26.0, developed by IBM Corp. in Armonk, NY.

## Results

The results, presented in Table 3, depict the mean values of proliferation for the different tested materials after 7 and 14 days. After one week of culture, there was no significant difference observed among the tested groups. However, after two weeks, TheraCal LC exhibited the highest proliferation values compared to the other tested materials.

**Table 3:** Mean and Standard deviation (SD) for Proliferation of different tested groups

		Control		Ca Hydroxide		Glass Ionomer		Theracal LC		*p*-value
		Mean	SD	Mean	SD	Mean	SD	Mean	SD	
PROLIFERATION	W1	96.4^ac^	2.7	100.6^a^	3.6	102.2^a^	10.8	103.7^a^	6.3	0.214 NS
	W2	96.6^bc^	3.1	102.2^ab^	4.3	105.0^ab^	10.2	109.3^a^	7.2	0.012*
*p*-value		0.887 NS		0.479 NS		0.434 NS		0.058 NS		

^a,b,c^Different letters within each row indicates significant difference

*NS* Non-significant, * = significant

**Fig. 2:**
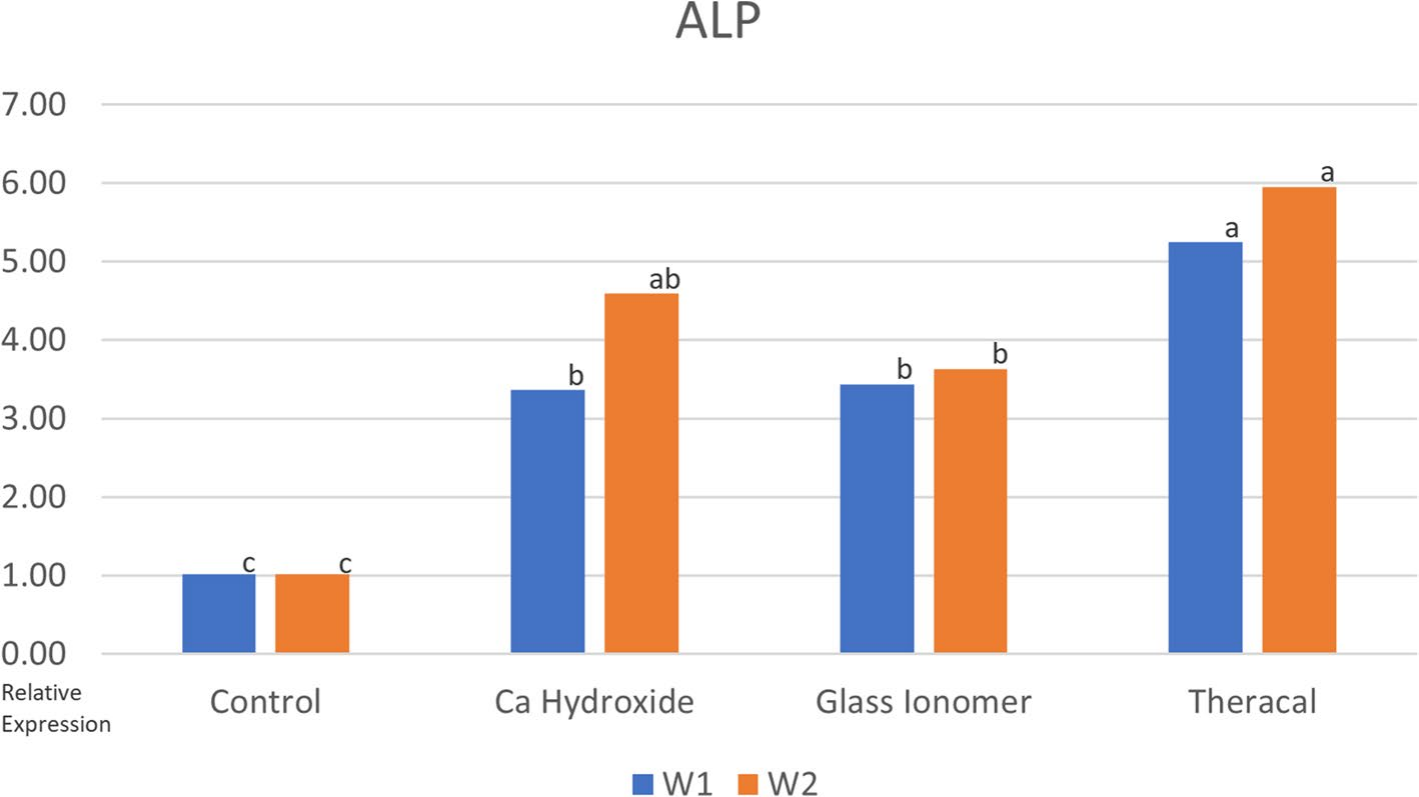
Bar chart showing the mean ALP for tested group. Different letters above bars indicates significant difference

The results of the present study indicate a significant increase in ALP enzyme activity in the TheraCal LC, Calcium hydroxide, and Glass ionomer groups compared to the control group after 7 days. Among these groups, TheraCal LC exhibited the highest increase in ALP enzyme activity. However, after 14 days, the increase in ALP enzyme activity in the Calcium hydroxide group was comparable to that of TheraCal LC, with no significant difference observed between the two materials. These findings are illustrated in (Fig. 2).

**Fig. 3:**
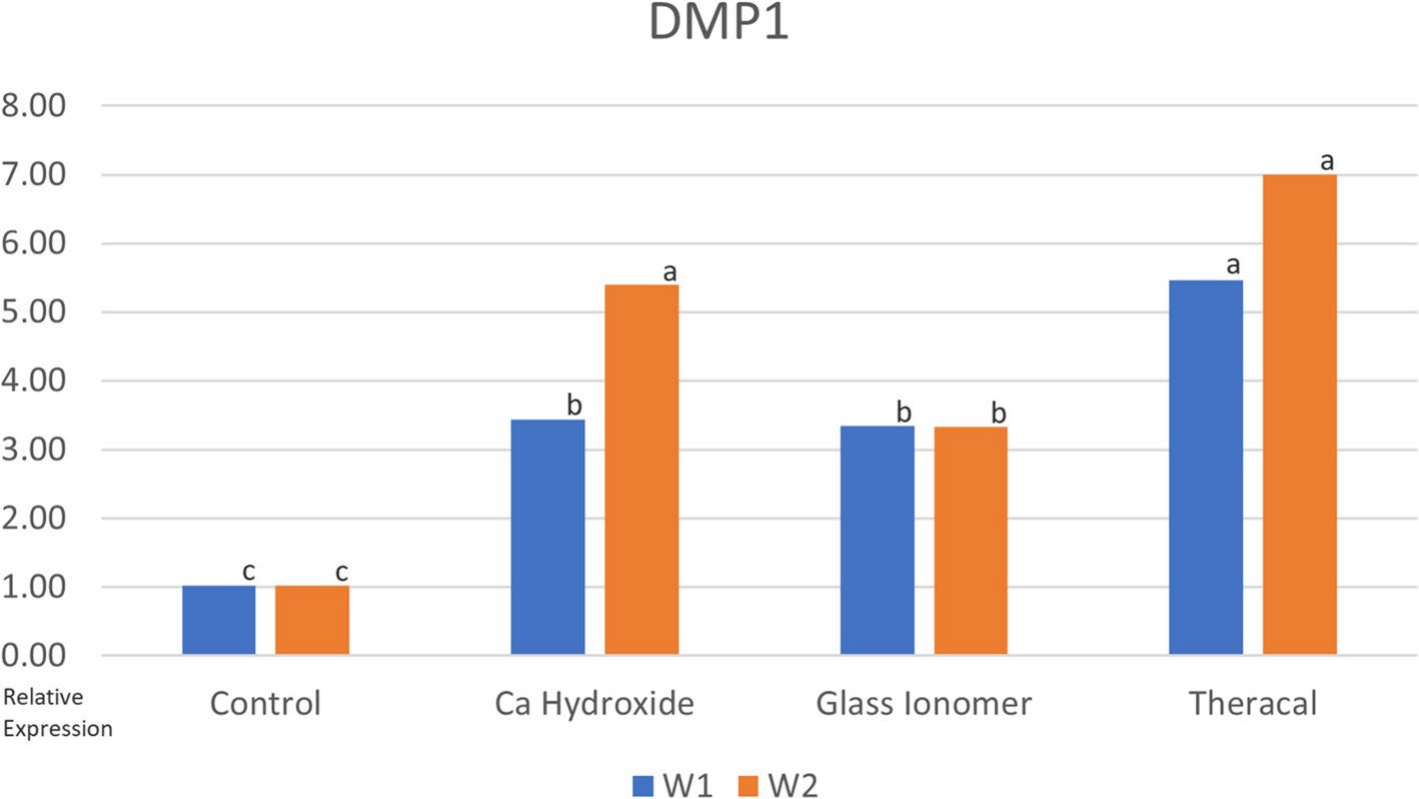
Bar chart showing the mean DMP1 for tested group. Different letters above bars indicates significant difference

The three materials used showed an increase in DMP-1 gene expression compared to the control group. This increase was found to be statistically significant, with the TheraCal LC being significantly higher than all other materials after 7 days of culture. But after 14 days it was only significant to the control and GI group, and that Ca(OH)_2_ showed comparable results to that of TheraCal LC (Fig. 3).

**Fig. 4:**
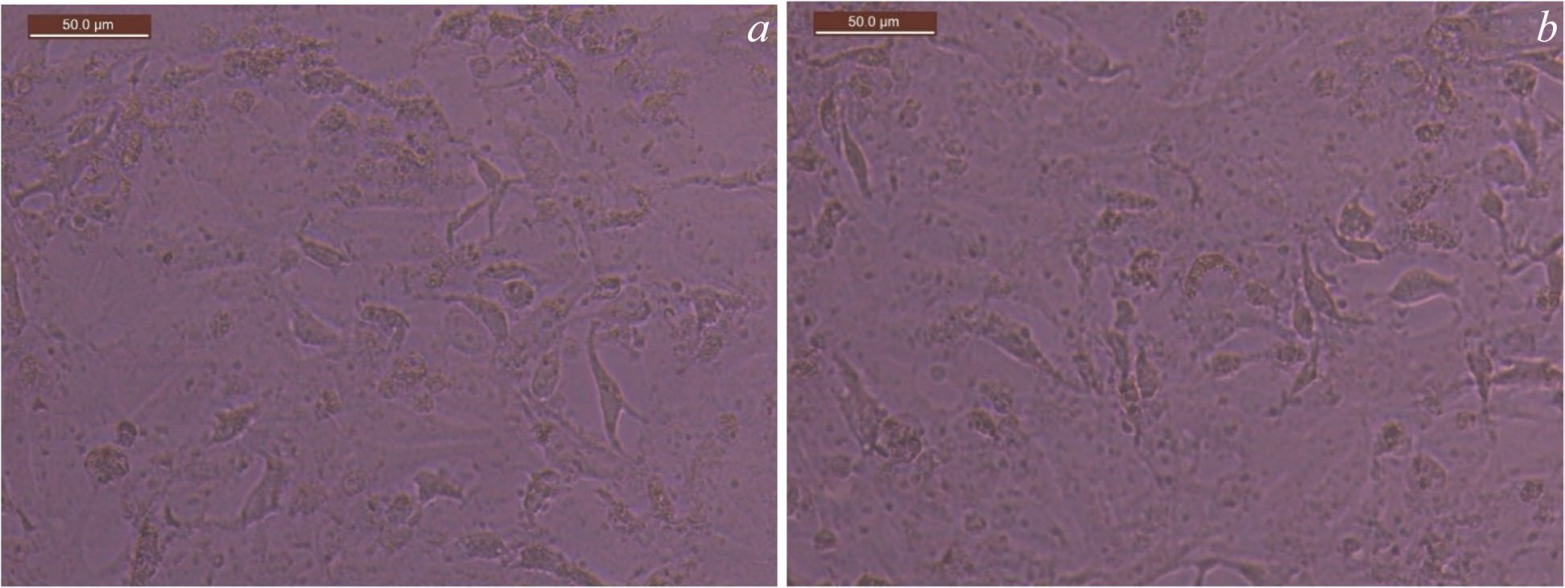
**a** Photomicrograph showing undifferentiated cells of the control group (day 7), **b** Photomicrograph showing undifferentiated cells of the control group (day 14)

**Fig. 5:**
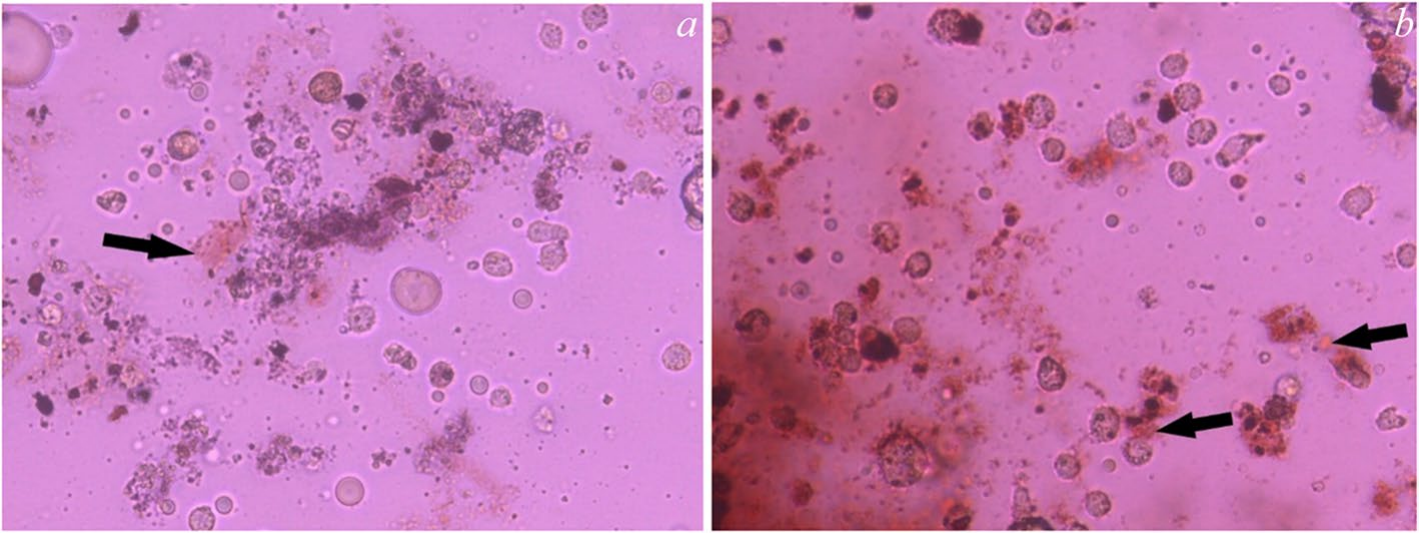
**a** Differentiated cells (day 7) subjected to Ca(OH)_2_ stained with Alizarin Red showing calcific deposits on its cell borders.( 100 × magnification). **b** Differentiated cells (day 14) subjected to Ca(OH)_2_ stained with Alizarin Red showing increase calcific deposits inside the cells and at the cell boundary. (100 × magnification)

**Fig. 6:**
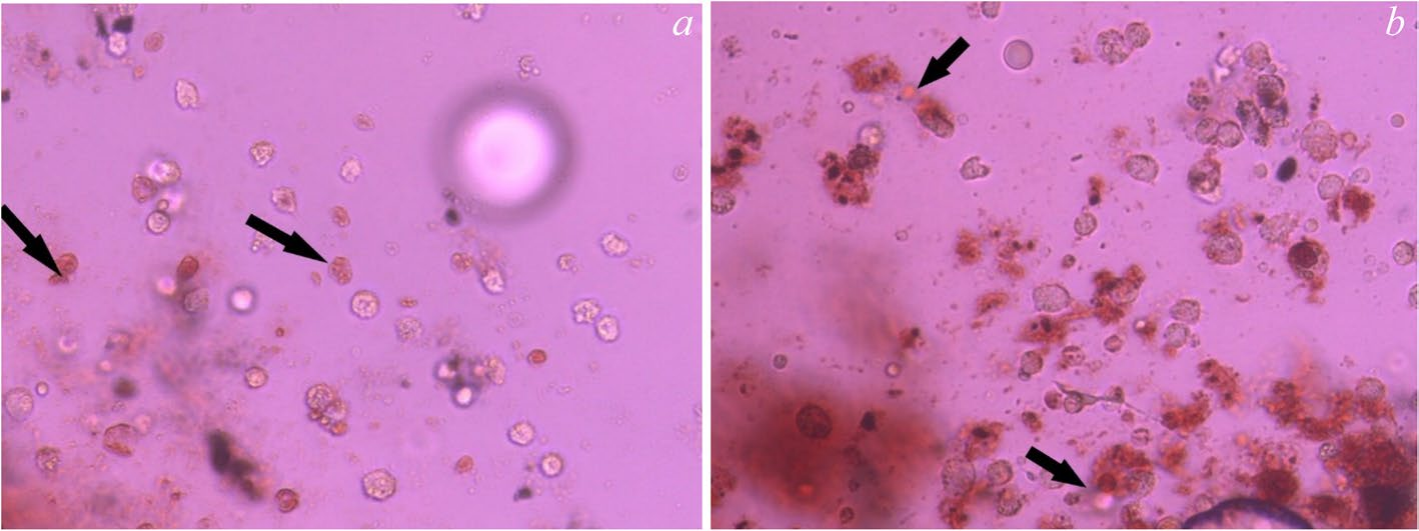
**a** Differentiated cells (day 7) subjected to Glass ionomer (group III) stained with Alizarin Red showing calcific deposits on its cell borders.(100 × magnification, **b** Differentiated cells (day 14) subjected to Glass ionomer stained with Alizarin Red showing increase calcific deposits inside the cells and at the cell boundary.( 100 × magnification)

**Fig. 7:**
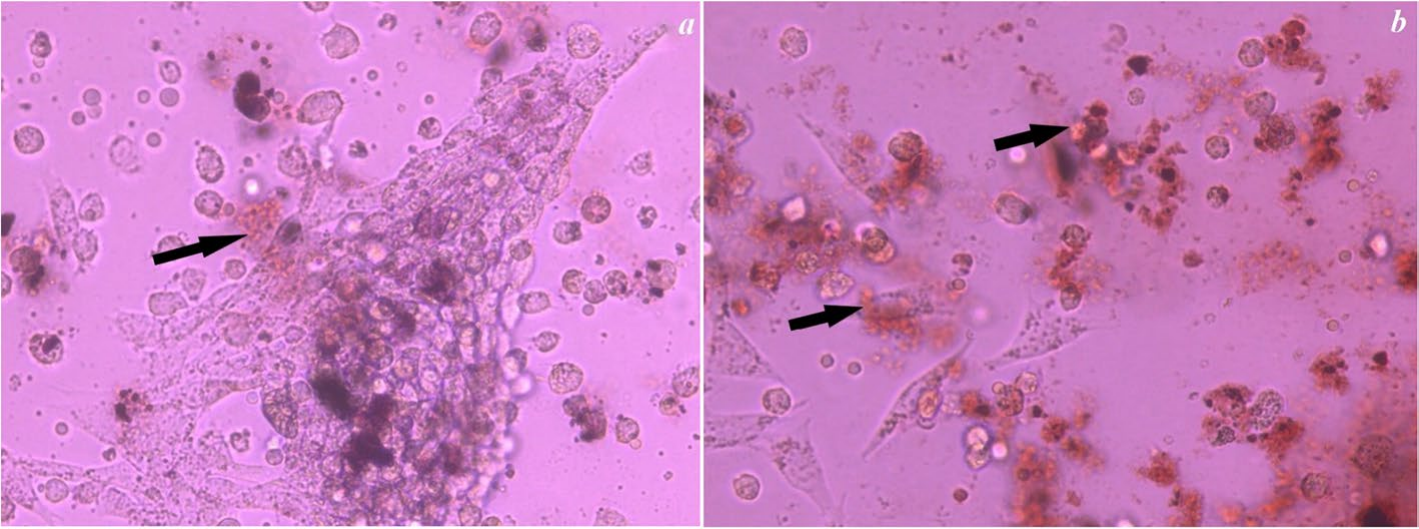
**a** Photomicrograph showing differentiated cells (day 7) subjected to resin modified calcium silicate (TheraCal LC) (group IV) stained with Alizarin Red showing calcific deposits on its cell borders. (100 × magnification), **b** (Day 14) showing increase calcific deposits inside the cells and at the cell boundary. ( 100 × magnification)

The Alizarin Red S Staining assay in Figs. 4, 5, 6 and 7 showed the control group in Fig. 4a, b and revealed the presence of calcific deposits along the cell borders of Stem cells treated with Calcium hydroxide (CaoH2) Fig. 5a, Glass Ionomer Fig. 6a, and light-cured resin modified Calcium silicate (TheraCal LC) Fig. 7a after 7 days. Additionally, after 14 days, there was an increase in calcific deposits both inside the cells and at the cell boundary in Stem cells exposed to CaoH2 Fig. 5b, Glass Ionomer Fig. 6b, and light-cured resin modified Calcium silicate (TheraCal LC) Fig. 7b. The corresponding visual representation of these findings can be observed in Photomicrograph figures.

## Discussion

This study underscores the importance of utilizing stem cells sourced from human exfoliated deciduous teeth (SHED) in pulp and dentin regeneration. It highlights their power to proliferate and differentiate, leading to the formation of calcific bridges, which may serve to provide added protection to pulp tissues.

To ensure the quality of the isolated stem cells, only sound deciduous teeth with at least one-third of the root intact were used in the study. Carious teeth or teeth with any pathological conditions were excluded as they can negatively impact the success of SHED isolation [24]. The age range of children from 7 to 12 years was selected, as this corresponds to the normal shedding period of deciduous teeth. Patients without any systemic diseases that may affect stem cell proliferation or differentiation potential were included [25].

The study emphasizes the importance of proper collection and transportation of the tooth samples. While the extraction technique is not the primary factor affecting stem cell isolation, the storage and transport conditions significantly impact the viability of the living pulp and, consequently, the survival of stem cells [26].

Regarding the experimental results, the study found that SHED exhibited a similar proliferation rate to Calcium hydroxide, Glass ionomer, and TheraCal LC compared to the control group after a 7-day incubation period. However, after further incubation for 14 days, TheraCal LC demonstrated a significant increase in SHED proliferation compared to the control group. Thus, the null hypothesis of the study was rejected. The increase in proliferation observed with Glass ionomer and Calcium hydroxide was not statistically significant. These findings align with a previous study by Kim et al. (2020), which reported that TheraCal LC exhibited better biocompatibility and bioactivity compared to the control group and even Calcium hydroxide [27]. Overall, the study highlights the potential of TheraCal LC in promoting SHED proliferation and suggests its favorable biocompatibility and bioactivity compared to other tested materials.

The observation that the proliferation rate with Glass ionomer cement (GIC) was not significantly high raises caution regarding its usage in restorative dentistry, especially when in close proximity to pulp tissue. This indicates that GICs may not be the most suitable choice for capping materials, as they need to not only be biocompatible and support stem cell proliferation but also facilitate their differentiation.

In terms of ALP enzyme activity, the results of the present study showed a significant increase in the TheraCal LC, Calcium hydroxide, and Glass ionomer groups compared to the control group after 7 days. Among these groups, TheraCal LC demonstrated the highest increase. However, after 14 days, the significant increase in ALP enzyme activity seen with Calcium hydroxide was comparable to that of TheraCal LC, with no significant difference between the two materials. Another study also supported these findings, highlighting the low stem cell differentiation and ALP enzyme activity in specimens using Glass ionomer cement [28].

DMP-1 (Dentin Matrix Protein 1) is a gene involved in early odontoblast differentiation and plays a crucial role in the organization of collagen matrix and dentin mineralization [29]. In this study, all three materials resulted in an increase in DMP-1 gene expression compared to the control group. This increase was found to be statistically significant, with TheraCal LC exhibiting significantly higher expression than all other materials after 7 days. However, after 14 days, the significant difference was observed only between the control and Glass ionomer groups, while Calcium hydroxide showed comparable results to TheraCal LC. This finding aligns with a study by Araújo et al. in 2018, which demonstrated a gradual increase in DMP-1 gene expression in the Calcium hydroxide group over time [30].

To assess the presence of calcific deposits both inside and outside odontogenic lineage cells, Alizarin Red stain was employed as a biochemical assay after 7 and 14 days of culture. After 7 days, the staining revealed the formation of calcific deposits with all tested materials compared to the control group. Furthermore, after 14 days, calcific deposits inside and at the cell boundaries increased with all the materials. These findings indicate the ability of all tested materials to induce favorable differentiation of SHEDs.

Overall, the study highlights the differential effects of various materials on proliferation, ALP enzyme activity, gene expression, and calcific deposit formation in SHEDs. It emphasizes the potential of TheraCal LC in promoting stem cell proliferation, ALP enzyme activity, and DMP-1 gene expression, which can encourage the use of TheraCal LC in clinical practice. while also demonstrating the capability of all tested materials to induce favorable differentiation in SHEDs.

This study demonstrates several strengths. The most important of them is that it incorporates three widely employed materials in routine dental practice. Additionally, it harnesses the potential of SHEDs, an exceedingly promising category of stem cells. Furthermore, the study adopts a multifaceted approach to differentiation assessment, encompassing the measurement of ALP and DMP 1, alongside morphological evaluation utilizing the Alizarin Red S stain.

However, certain limitations should be acknowledged. The culture duration was relatively brief, spanning only 14 days. Moreover, the study solely took place in an in vitro setting, lacking the dynamic oral conditions that would be encountered in vivo.

## Conclusion

SHEDs possess the potential to differentiate into stable odontoblastic-like cells, making them a valuable source for regenerative approaches. This capability provides an alternative solution for preserving teeth with compromised structural integrity.

The results of the study indicate that the TheraCal LC group exhibited higher proliferation rates, ALP activity, and DMP-1 gene expression compared to the other groups. These findings suggest that TheraCal LC promotes superior odontoblastic differentiation when compared to the other tested materials.

### Supplementary Information

**Additional file 1.**

## Data Availability

The datasets used and/or analysed during the current study available from the corresponding author on reasonable request.

## Abbreviations

ALP: Alkaline phosphatase
Ca(OH)_2_: Calcium hydroxide
DFSC: Dental follicle stem cells
DMEM: Dulbecco’s modified eagle’s medium
DMP: Dentin matrix protein
DPSCs: Dental pulp stem cells
FBS: Fetal bovine serum
GIC: Glass ionomer cement
GMSCs: Gingival derived stem cells
HCSCs: Hydraulic calcium silicate– based cements
IPT: Indirect pulp therapy
MTT: 3-[4, 5-Dimethylthiazol-2yl]-2, 5-diphenyl-tetrazolium bromide
PBS: Phosphate buffered saline
PDLSCs: Periodontal ligament stem cells
QRT-PCR: Quantitative real time -polymerase chain reaction
SCAPs: Stem cells from the apical papilla
SHEDs: Stem cells from human exfoliated deciduous teeth
